# Comparative analysis of endoscopic-assisted burr hole craniostomy and two burr hole craniostomy in the treatment of septated chronic subdural hematoma

**DOI:** 10.3389/fneur.2025.1540877

**Published:** 2025-04-01

**Authors:** Yaqing An, Hongbo Cheng, Xiaoliang Wang, Yijiao Men, Jiegang Yu, Gengshen Zhang, Jiaming Li, Tao Zuo, Baohai Yu, Jianliang Wu, Yang Wu

**Affiliations:** ^1^Emergency Department, The Second Hospital of Hebei Medical University, Shijiazhuang, China; ^2^Department of Neurosurgery, The Second Hospital of Hebei Medical University, Shijiazhuang, China

**Keywords:** septated chronic subdural hematoma, endoscopic-assisted surgery, two burr hole craniostomy, recurrence, outcome

## Abstract

**Objective:**

To conduct a retrospective comparative analysis of endoscopic-assisted burr hole craniostomy (EBHC) vs. two burr hole craniostomy (TBHC) in the management of septated chronic subdural hematoma (sCSDH).

**Methods:**

This study employed a retrospective cohort design, encompassing 87 patients diagnosed with sCSDH who were admitted for EBHC or TBHC between January 2018 and December 2023. Among these patients, 48 underwent EBHC, while 39 received TBHC. The primary outcome measure was the recurrence rate within 6 months following surgery. Secondary outcomes included clinical outcomes at discharge and 6 months, duration of hospitalization, and duration of drainage tube placement.

**Results:**

The recurrence rates were comparable between EBHC and TBHC groups (12.5% vs. 10.3%, *p* = 0.742). However, the mean operative time was significantly longer for the EBHC group, averaging 103.56 ± 20.93 min, in contrast to the TBHC group, which averaged 50.77 ± 12.40 min (*p* < 0.001). Additionally, the mean placement time for the drainage tube was significantly shorter in the EBHC group (18.66 ± 5.89 h) compared to the TBHC group (55.87 ± 23.03 h, *p* < 0.001). Furthermore, the mean length of hospital stay was notably reduced for the EBHC group (6.02 ± 1.68 days) compared to the TBHC group (4.66 ± 1.79 days, *p* < 0.001). There were no significant differences in mortality rates, complication rates, MRS scores, GCS scores, or the presence of gross focal neurological deficits postoperatively between the groups.

**Conclusion:**

TBHC is an effective intervention for sCSDH, offering a less invasive alternative with shorter operative duration and comparable recurrence rates to EBHC. Nonetheless, the efficacy of this approach requires further validation through large, multicenter studies.

## Introduction

Chronic subdural hematoma (CSDH) is a common neurosurgical entity, frequently encountered in clinical practice, particularly among elderly populations (1). In a subset of cases, CSDH exhibits a distinct pathological architecture characterized by fibrin septations and thickened inner membranes, demarcating these lesions as septated chronic subdural hematoma (sCSDH) (2). The compartmentalization inherent to sCSDH disrupts hematoma fluid dynamics, impeding effective drainage and elevating the risk of recurrence (3). While single burr hole craniostomy (SBHC) remains a standard intervention for non-septated CSDH due to its simplicity and efficacy, its utility in sCSDH is limited by the inability to address multiloculated cavities and persistent septal barriers (4). Craniotomy, though historically used, is associated with higher morbidity and longer recovery, particularly in elderly patients (5). Consequently, sCSDH presents a unique therapeutic challenge (6, 7).

In an evidence-based review of treatment modalities for sCSDH, the selection of two-burr hole craniostomy (TBHC) and endoscopic-assisted burr hole craniostomy (EBHC) for this study was based on their distinct advantages in addressing the unique challenges posed by septated chronic subdural hematoma (sCSDH) (2). TBHC allows for bidirectional drainage, reducing recurrence risk by addressing septations through irrigation, and remains the standard approach in many institutions because of its simplicity and lower technical demands. Recent study evaluated the number of burr holes as a predictor for recurrence, concluding that treatment of sCSDH through a single burr hole is associated with a markedly higher recurrence rate, and extended hospital stays compared to the TBHC (8). Other study determined that two-burr hole approach, combined with irrigation and drainage, offers the most favorable cure-to-complication ratio (2, 4). In addition to the previously mentioned two burr hole drainage techniques, endoscopic-assisted evacuation of subdural collections has also been introduced (9, 10). EBHC combines burr hole evacuation of chronic subdural hematomas with endoscopic visualization of the subdural space, enabling precise inspection and targeted resection of septa and neomembranes (11). Numerous neurosurgeons believe that endoscopic surgery may represent an appropriate intervention for sCSDH, as it is associated with reduced trauma and a lower recurrence rate (12, 13). Several studies have established that endoscopic surgery surpasses traditional techniques in the management of sCSDH (14–17). Despite the extensive research supporting the advantages of both surgical techniques in the treatment of sCSDH, no one can definitively claim which is the best surgical technique. The aim of this investigation was to conduct a retrospective comparison of EBHC and TBHC in the treatment of sCSDH.

## Materials and methods

The study design is a retrospective cohort comparison study. We retrospectively collected and analyzed data from patients diagnosed with sCSDH admitted to our institution between January 2018 and December 2023. All patients who underwent either EBHC or TBHC were included in the analysis. Patients were excluded if they (a) underwent craniotomy, (b) received conservative (medical) treatment, or (c) presented with recurrent subdural hematomas. Among the 87 patients, 48 were treated with EBHC, while 39 received TBHC. All procedures were conducted by a senior neurosurgical team with at least 3 years of experience in TBHC or EBHC. Following a computed tomography (CT) diagnosis and clinical evaluation, the surgical procedures were conducted as follows.

### TBHC group

**Figure 1:**
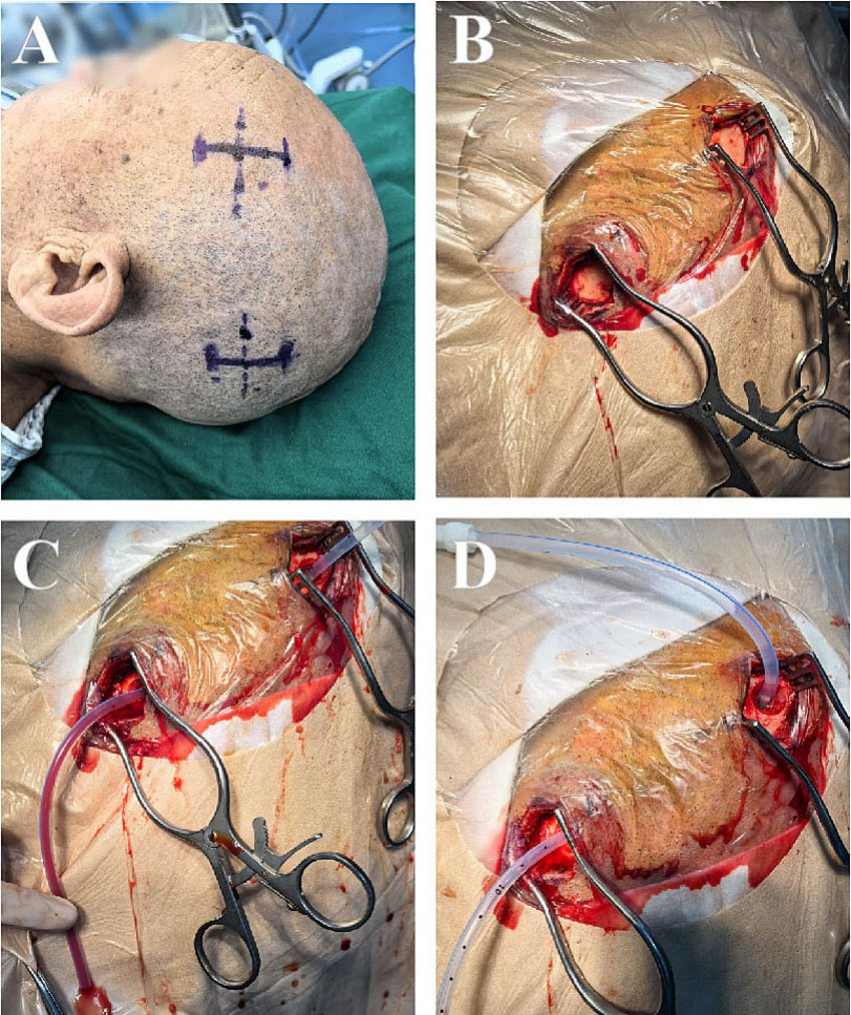
The surgical procedure for TBHC: identification of the surgical incision **(A)**, exposure of the surgical bone window **(B)**, irrigation with warm isotonic saline **(C)**, evacuation of solid hematoma clots until the sodium chloride solution appeared clear **(D)**.

**Figure 2:**
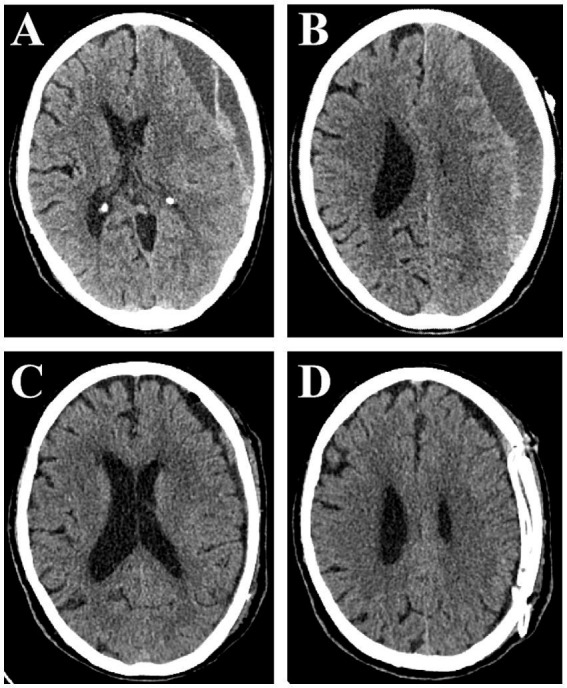
Computed tomography scan showing septated chronic subdural hematoma on the left side. Small red arrows indicate SCSH with two clear compartments **(A,B)**. Early postoperative scan showing significant clearance of hematoma, regression of midline shift, and slight intracranial pneumatosis **(C,D)**.

TBHC was chosen for patients with less complex septations suitable for irrigation via dual burr holes. Patients on long-term anticoagulants or with significant comorbidities (e.g., ischemic heart disease) were also preferred for TBHC under local anesthesia. All patients who underwent this surgical procedure received infiltration with local scalp anesthesia, comprising a solution of 1% lidocaine combined with 1:100,000 epinephrine, mixed equally with 0.5% bupivacaine. Anesthesia personnel were on standby to provide sedation and monitoring. Each patient was positioned supine, with the head rotated approximately 60° toward the contralateral side. Surgical interventions involved the creation of two burr holes at the site of hematoma expansion. The surgeon performed a longitudinal incision, roughly 2.5 cm in length, over the lesion, retracted the skin, and subsequently stripped the periosteum prior to drilling. The dura mater was incised using a No. 15 curved blade, after which the dura edges were retracted. Simultaneously, the neurosurgeons executed the same procedure through additional burr holes. Two closed drainage system was subsequently established. The subdural membrane remained intact, except for those that were readily accessible through the burr holes. Ultimately, two flexible blunt drainage tubes were strategically positioned in the frontal and occipital directions of the subdural space to ensure the efficient drainage of the residual hematoma. Then, the hematoma cavity was irrigated with warm isotonic saline using a 50 mL syringe, facilitating the evacuation of any solid hematoma clots until the sodium chloride solution appeared clear. The burr holes were sealed with absorbable gelatin compressed sponge, and the skin was sutured in two layers (Figure 1). The drainage tubes were removed following the desiccation of the hematoma secretion and confirmation of adequate treatment via CT imaging (Figure 2).

### EBHC group

**Figure 3:**
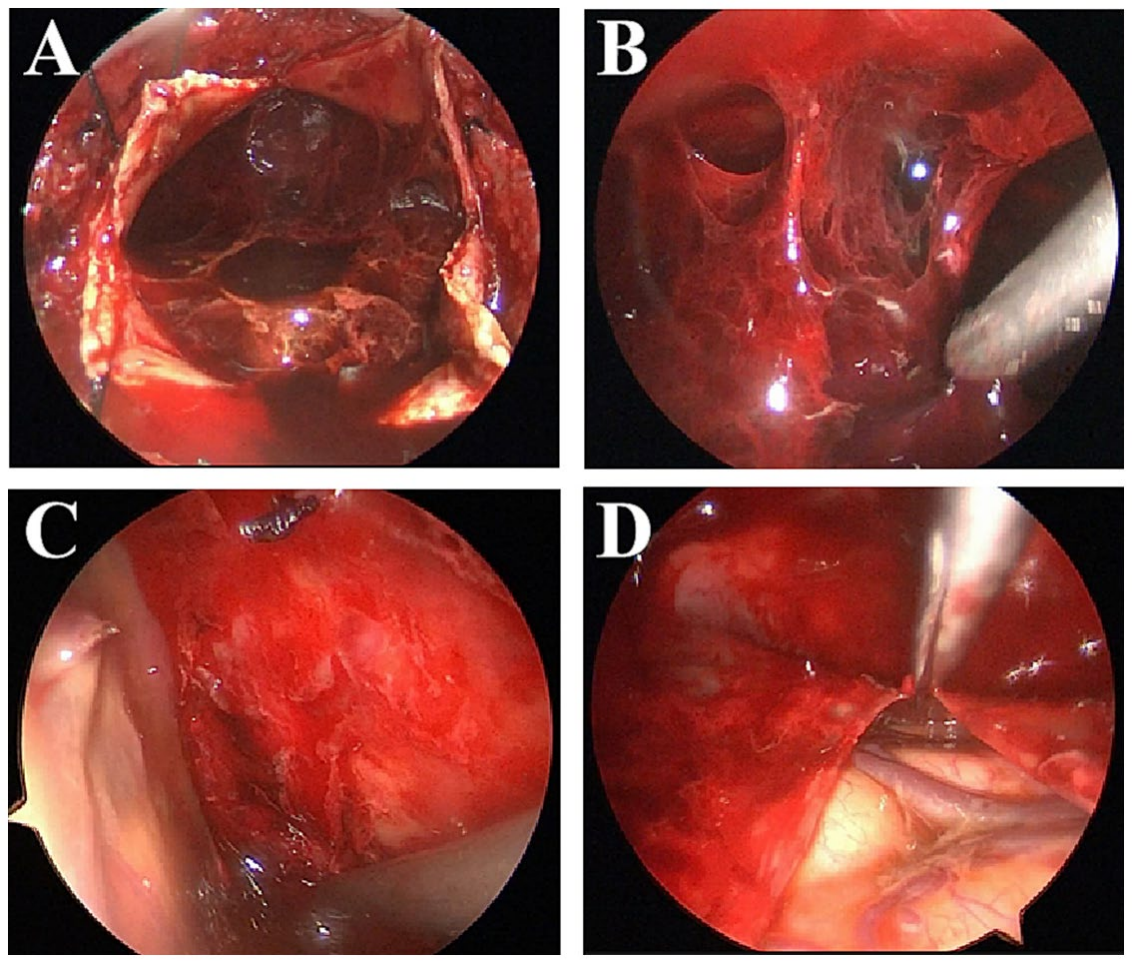
Various intraoperative endoscopic findings: multiple septations **(A,B)**, cortical bridging veins and after hematoma flushing is complete **(C)**, Excision of the internal membrane of the hematoma **(D)**.

**Figure 4:**
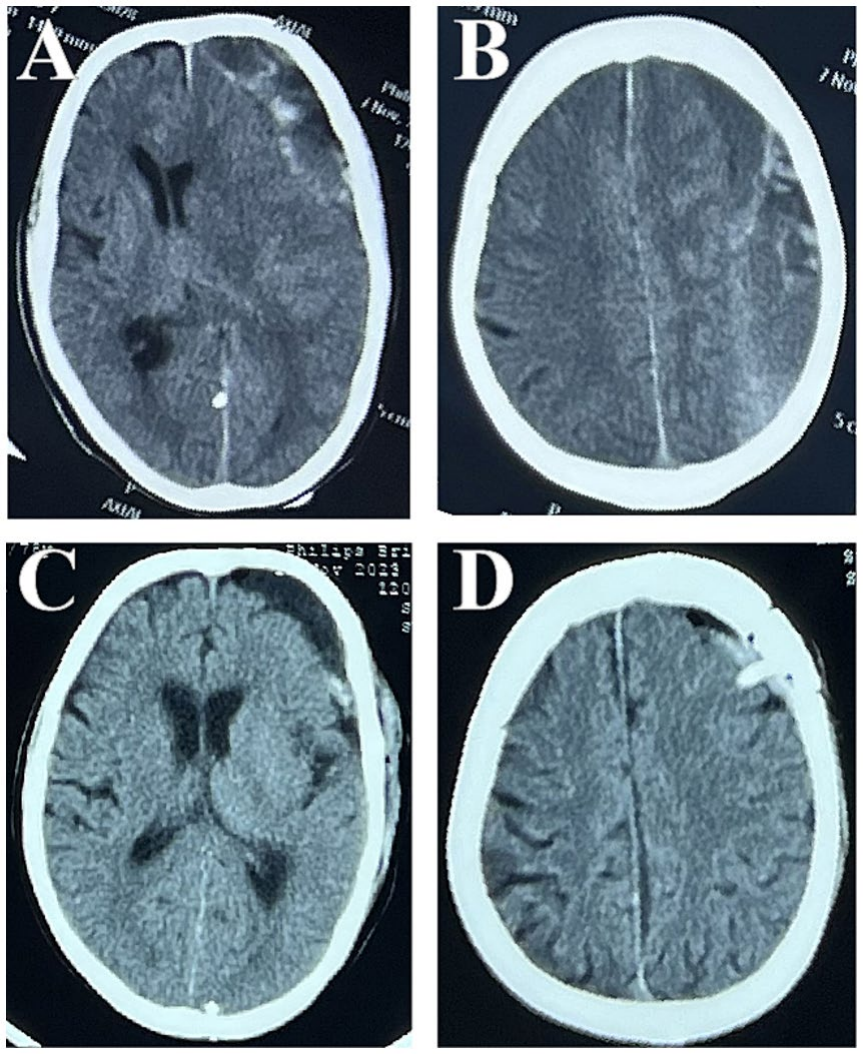
Preoperative CT examination showed a large left-sided front temporal occipitoparietal sCSDH with prominent midline shift; the subdural cavity contained several fibrous septa **(A,B)**. Postoperative CT shows the evacuated haematoma with expected fluid **(C,D)**.

Patients exhibiting the following characteristics were preferentially assigned to EBHC group: Radiologically confirmed thick septations or neomembranes (identified via preoperative MRI/CT), which required direct visualization for targeted septotomy; Active neovessels (detected on MRI) necessitating endoscopic coagulation; Complex hematoma architecture, such as calcified compartments. All patients who underwent this surgical procedure were placed under intratracheal general anesthesia. Each patient was positioned supine, with the head rotated approximately 30° toward the side contralateral to the lesion. The surgeon created a cranial bone flap approximately 2.5 cm in diameter over the lesion, distal to the long axis of the hematoma cavity. The dura mater was incised using a No. 15 curved blade, and the edges were retracted and secured with 3–0 Nurolon sutures. The outer membrane of the CSDH was subsequently incised. A rigid endoscope with a 30° angle and 4 mm diameter (Karl Storz, Tuttlingen, Germany) was inserted into the CSDH cavity, and suction was employed to remove visible hematoma remnants. Blood clots of varying densities were evacuated. Utilizing the endoscope, we identified any neovessels previously detected via MRI and coagulated and ligated them as necessary. Finally, we thoroughly irrigated the subdural space with saline solution and established a closed drainage system (Figures 3, 4).

We documented essential patient demographics, preoperative neurological impairments, social histories, medical histories, medication regimens, and the characteristics of the hematoma as observed on CT scans. The volumes of hematomas were calculated using the XYZ/2 method (18). Furthermore, we compared surgical conditions, operative duration, drainage tube placement times, and lengths of hospital stays associated with neurosurgical interventions. Patients were discharged to their homes once they no longer required specialized neurosurgical care. All patients were closely monitored in accordance with established research protocols, with follow-up assessments encompassing progressive CT scans, Modified Rankin Scale (MRS), Glasgow Coma Scale (GCS), and analyses of postoperative recurrence, morbidity, and mortality.

The primary outcome measure was the recurrence rate, defined as the frequency of reoperation necessitated by recurrent CSDH in patients previously treated with EBHC or TBHC. Recurrence was characterized by the emergence of symptoms and signs attributable to an ipsilateral hematoma, as evidenced by a CT scan within 6 months following the initial drainage procedure. Reoperation was warranted in cases of any deterioration in original neurological deficits, a decline GCS or MRS scores, the emergence of new focal neurological deficits, or evidence of increased size of the CSDH on CT during the follow-up period. Secondary outcome measures included clinical outcome at discharge, and at 6 months; the duration of hospitalization for neurosurgery; and the duration of drainage tube placement. Postoperative outcomes were assessed by blinded neuroradiologists and neurologists uninvolved in surgical decision-making, minimizing observer bias.

Statistical analyses were conducted using the Chi-square test or Fisher’s Exact Test for categorical frequencies. For continuous variables, independent t-tests were employed. A significance level of 5% was established, and all statistical evaluations were carried out using SPSS software version 19.0.

## Results

The demographic characteristics of the studied patients are summarized in Table 1. The cohort consisted of 87 patients, with a median age of 73.65 ± 4.99 years, including 62 males (72.8%) and 25 females (28.73%). Among these patients, 48 (55.17%) were treated with EBHC, while 39 (44.82%) underwent TBHC. Of the 87 patients, 9 (10.34%) required reoperation due to recurrence. The mean Markwalder scale score upon admission was 1.85 ± 0.77, and the average initial hematoma thickness was 17.51 ± 4.85 mm. The average length of hospital stay was 5.41 ± 1.85 days. Complications (excluding recurrences) were observed in 17 patients (19.54%), and 3 patients succumbed during the 6-month postoperative follow-up, primarily due to pneumonia, pulmonary embolism, and multiple organ failure. Regarding medical history, 45 patients (51.72%) were previously healthy, while 22 (25.28%) had diabetes, 31 (35.63%) suffered from hypertension, 23 (26.43%) were on antiplatelet therapy, 19 (21.83%) received anticoagulation, 9 (10.34%) had a history of cerebrovascular accidents, and 15 (17.24%) presented with ischemic heart disease. The hematoma was located on the left side in 46 patients (52.87%), on the right side in 35 (40.23%), and bilaterally in 6 (6.89%) patients.

**Table 1:** Summary of demographic data of studied patients.

Characteristics	Total patients = 87
	Mean ± SD/*n* (%)
Mean age (years)	73.65 (4.99)
Gender	
Male	62 (71.26%)
Female	25 (28.73%)
Total no. of haematomas with EBHC	48 (55.17%)
Total no. of haematomas with TBHC	39 (44.82%)
Total no. of operative recurrences	9 (10.34%)
Initial Markwalder scale	1.85 (0.77)
Hospitalization length (days)	5.41 (1.85)
Thickness of initial haematoma (mm)	17.51 (4.85)
Mortality	3 (3.4%)
Complication (other than recurrences)	17 (19.54%)
Medical history	
No	45 (51.72%)
Diabetes	22 (25.28%)
Hypertension	31 (35.63%)
Antiplatelet	23 (26.43%)
Anticoagulation	19 (21.83%)
Cerebrovascular accident	9 (10.34%)
Ischemic heart disease	15 (17.24%)
sCSDh sites	
Left	46 (52.87%)
Right	35 (40.23%)
Bilateral	6 (6.89%)

Table 2 presents the clinical features of both groups. The mean age of patients in the EBHC group was 73.75 years, compared to 73.51 years in the TBHC group (*p* = 0.827). The majority of patients were male, comprising 75.0% in the EBHC group and 66.7% in the TBHC group. The most prevalent symptoms included gait disturbances, limb weakness, headaches, drowsiness or coma, aphasia, seizures, incontinence, and vomiting, with no significant differences observed between the two groups. Additionally, no discrepancies were noted in the rates of smoking, alcohol consumption, or premorbid mobility. In the EBHC group, 7 (14.6%) and 5 (10.4%) patients had received antiplatelet and anticoagulant therapy, respectively, compared to 16 (41.0%) and 14 (35.9%) in the TBHC group (*p* = 0.005, *p* = 0.004). Furthermore, 5 of 48 patients (10.4%) in the EBHC group and 11 of 39 patients (28.2%) in the TBHC group had a documented history of ischemic heart disease (*p* = 0.033). The Markwalder scores were 1.77 ± 0.83 for the EBHC group and 1.94 ± 0.68 for the TBHC group (*p* = 0.277).

**Table 2:** Demographic and clinical features of studied patients in the two groups [Mean ± SD/*n* (%)].

Variables	EBHC (*n* = 48)	TBHC (*n* = 39)	*p*
Age (years)	73.75 ± 4.80	73.51 ± 5.27	0.827
Male gender	36/48 (75.0%)	26/39 (66.7%)	0.393
Premorbid mobility			
Independent	11/48 (22.9%)	5/39 (12.8%)	0.227
Stick	7/48 (14.6%)	8/39 (20.5%)	0.467
Frame	10/48 (20.8%)	13/39 (33.3%)	0.189
Wheelchair	11/48 (22.9%)	7/39 (17.9%)	0.569
Bed-bound	9/48 (18.8%)	6/39 (15.4%)	0.679
Preoperative neurological dysfunction			
Gait disturbance	15/48 (31.3%)	14/39 (35.9%)	0.647
Limb weakness	16/48 (33.3%)	15/39 (38.5%)	0.619
Headache	19/48 (39.6%)	17/39 (43.6%)	0.706
Drowsiness or coma	17/48 (35.4%)	15/39 (38.5%)	0.770
Aphasic disorder	16/48 (33.3%)	19/39 (48.7%)	0146
Seizure	5/48 (33.3%)	7/39 (48.7%)	0.311
Incontinence	17/48 (35.4%)	8/39 (20.5%)	0.127
Vomiting	9/48 (18.8%)	12/39 (30.8%)	0.193
Social history			
Drinker	23/48 (47.9%)	18/39 (46.2%)	0.870
Smoker	19/48 (39.6%)	11/39 (28.2%)	0.267
Medical history			
Diabetes	8/48 (16.7%)	12/39 (30.8%)	0.120
Hypertension	13/48 (27.1%)	18/39 (46.2%)	0.065
Antiplatelet	7/48 (14.6%)	16/39 (41.0%)	<0.01
Anticoagulation	5/48 (10.4%)	14/39 (35.9%)	<0.01
Cerebrovascular accident	4/48 (8.3%)	7/39 (17.9%)	0.309
Ischemic heart disease	5/48 (10.4%)	11/39 (28.2%)	<0.05
Markwalder score	1.77 ± 0.83	1.94 ± 0.68	0.277

As illustrated in the CT scan results (Table 3), the majority of patients exhibited mixed-density hematomas, and the mean hematoma volume was comparable across the two groups (81.62 ± 26.42 mL for EBHC vs. 76.45 ± 42.76 mL for TBHC, *p* = 0.511). No significant differences were observed in the localization and distribution of hematomas. Additionally, the mean midline shift measured 9.13 mm for EBHC and 9.22 mm for TBHC (*p* = 0.827), while the maximal width of the initial hematoma was recorded at 16.91 mm for EBHC and 18.25 mm for TBHC (*p* = 0.202). The surgical conditions are detailed in Table 4, where the mean operative time was significantly longer for the EBHC group (103.56 ± 20.93 min) in comparison to the TBHC group (50.77 ± 12.40 min; *p* < 0.001). Additionally, no statistically significant differences were noted between the two groups with respect to other parameters.

**Table 3:** CT features on admission.

Variables	TBHC (*n* = 48)	TBHC (*n* = 39)	*p*
Localization			
Left	24/48 (50.0%)	22/39 (56.4%)	0.551
Right	20/48 (41.7%)	15/39 (38.5%)	0.762
Bilateral	4/48 (8.3%)	2/39 (5.1%)	0.872
Density			
Hypodense	15/48 (31.3%)	9/39 (23.1%)	0.396
Isodense	13/48 (27.1%)	12/39 (30.8%)	0.706
Mixed	20/48 (41.7%)	17/39 (46.2%)	0.675
Mean distribution			
Frontal	23/48 (47.9%)	15/39 (38.5%)	0.377
Temporal	34/48 (70.8%)	25/39 (64.1%)	0.504
Parietal	23/48 (47.9%)	20/39 (51.3%)	0.755
Occipital	24/48 (50.0%)	14/39 (35.9%)	0.187
Mean hematoma volume (mL)	81.62 ± 26.42	76.45 ± 42.76	0.511
Mean midline shift (mm)	9.13 ± 1.87	9.22 ± 2.21	0.827
Maximal width of initial hematoma	16.91 ± 4.66	18.25 ± 5.04	0.202

**Table 4:** Surgical condition.

Variables	ES (*n* = 48)	TBHC (*n* = 39)	*p*
Mean operative time (minutes)	103.56 ± 20.93	50.77 ± 12.40	<0.001
Subdural fluid pressure			
Low	11/48 (22.9%)	10/39 (25.6%)	0.768
Medium	15/48 (31.3%)	12/39 (30.8%)	0.962
High	15/48 (31.3%)	13/39 (33.3%)	0.836
Very high	7/48 (14.6%)	4/39 (10.3%)	0.780
Membrane			
Thin	9/48 (18.8%)	10/39 (25.6%)	0.439
Thick	26/48 (54.2%)	17/39 (43.6%)	0.326
Very thick	13/48 (27.1%)	12/39 (30.8%)	0.706
Subdural fluid			
Clear	6/48 (12.5%)	2/39 (5.1%)	0.418
Straw	9/48 (18.8%)	11/39 (28.2%)	0.297
Engine oil	10/48 (20.8%)	12/39 (30.8%)	0.289
Fresh blood	6/48 (12.5%)	1/39 (2.6%)	0.194
Mixture	17/48 (36.2%)	13/39 (33.3%)	0.783

As shown in the clinical outcomes (Table 5), the recurrence rates were comparable between the two groups, with 4 of 48 patients (8.3% [95% CI, 0.2–16.4]) in the EBHC group and 5 of 39 patients (12.8% [95% CI, 1.8–23.8]) in the TBHC group (*p* = 0.742). Medical consumption were significantly greater in the EBHC group, with a mean of 4.17 ± 0.83 (RMB × 10^4^; 95% CI, 3.92–4.41), compared to 1.55 ± 0.35 (RMB × 10^4^; 95% CI, 1.44–1.66) in the TBHC group (*p* < 0.001). The complication rate, excluding recurrences, was similar between the groups (*p* = 0.378), with complication rates of 11 (22.9%) and 6 (15.4%) observed in the EBHC and TBHC groups, respectively. Postoperatively, 8 patients treated with EBHC developed intracranial pneumatosis, compared to 3 in the TBHC group; In the EBHC group, two patients developed intracerebral hematomas, as did two patients in the TBHC group; Seizures were documented in two individuals following EBHC, compared to one individual in the TBHC group. Notably, one patient in the EBHC group experienced both postoperative intracranial pneumatosis and seizures. After treatment, all patient symptoms were significantly relieved or disappeared. At 30 days, mortality rates did not differ significantly between the EBHC group (2, 4.2%) and the TBHC group (1, 2.6%; *p* = 0.680). In the EBHC group, two patients died: one from myocardial infarction and the other from severe pneumonia, while in the TBHC group, one patient succumbed to heart failure. Furthermore, no significant differences were observed in the proportions of MRS scores ranging from 0 to 3 and the incidence of gross focal neurological deficits at the 6-month follow-up, as well as GCS scores at discharge. The average duration of drainage tube placement was markedly shorter in the EBHC group, at 18.66 ± 5.89 h (95% CI, 16.95–20.37), compared to 55.87 ± 23.03 h (95% CI, 48.41–63.34) in the TBHC group (*p* < 0.001). Additionally, the mean length of hospital stay was also significantly longer in the EBHC group, averaging 6.02 ± 1.68 days (95% CI, 5.53–6.51), compared to 4.66 ± 1.79 days (95% CI, 4.08–5.25) in the TBHC group (*p* < 0.001). The mean hematoma reduction rate was significantly greater in the EBHC group compared to the TBHC group, both at the first postoperative day (84.19% [95% CI, 81.86–86.52] vs. 64.63% [95% CI, 60.83–68.44], *p* < 0.001) and at discharge (94.69% [95% CI, 93.53–95.85] vs. 86.23% [95% CI, 84.79–87.66], *p* < 0.001).

**Table 5:** Primary and secondary outcome.

Variables	EBHC (*n* = 48)	TBHC (*n* = 39)	*p*
Recurrence	4/48 (8.3%)	5/39 (12.8%)	0.742
Mortality At 30 days	2/48 (4.2%)	1/39 (2.6%)	0.680
Complication (other than recurrences)	11/48 (22.9%)	6/39 (15.4%)	0.378
Medical consumption (RMB × 10^4^)	4.17 ± 0.83	1.55 ± 0.35	<0.001
Rankin (MRS 0–3) At 6 months	44/48 (91.7%)	36/39 (92.3%)	0.913
GCS (at discharge)	12.54 ± 1.75	13.10 ± 1.55	0.122
Gross focal neurological deficit At 6 months	15/48 (31.3%)	10/39 (25.6%)	0.565
Mean placement time of drainage tube (hours)	18.66 ± 5.89	55.87 ± 23.03	<0.001
Mean length of hospital stay for neurosurgery (days)	6.02 ± 1.68	4.66 ± 1.79	<0.001
Mean hematoma reduction rate (%)			
At first postoperative day	84.19 ± 8.01	64.63 ± 11.74	<0.001
At discharge	94.69 ± 3.98	86.23 ± 4.41	<0.001

## Discussion

sCSDH is a special type of CSDH exhibiting a multiseptated or multilayered appearance (13). In the cases of sCSDH, surgeons are faced with the following two challenges: (1) removing the compressing haematoma effectively and (2) eliminating the possibility of intraoperative recurrence (19–22). A principal complication arises from the division of the hematoma by neomembranes into compartments, which obstructs the drainage of hematoma fluid (13). Previous research demonstrated that an endoscopic technique, combined with a closed drainage system, is preferable to membranectomy (23). They successfully employed endoscopy and resection of neomembranes in treating 13 out of 14 patients who had undergone burr-hole drainage, with no instances of recurrence observed. Additionally, TBHC has gained substantial support in the literature as a prevalent surgical intervention for sCSDH, demonstrating a markedly lower postoperative recurrence rate, a reduced duration of hospital stay, and a diminished incidence of wound infections when compared to single burr hole procedures (2, 4). Although the aforementioned surgical approaches are widely regarded as yielding superior outcomes, there is a notable scarcity of published studies directly comparing the results of EBHC and TBHC for sCSDH. This study aims to address this gap.

### EBHC vs. TBHC

TBHC is frequently regarded as less effective than EBHC in the evacuation of hematomas for sCSDH, particularly in cases involving thick hematomas (12, 13). Nevertheless, our findings indicate that the surgical outcomes for sCSDH following EBHC and TBHC are comparable. Specifically, patients treated with EBHC exhibited a recurrence rate of 8.3%, compared to 12.8% for those undergoing TBHC, a difference that, though noteworthy, did not achieve statistical significance. Previous research concluded that in most instances of sCSDH, multiplicity does not imply multiple closed cavities, but rather that all hematoma cavities are interconnected through relatively wide channels (24). This prior research insight effectively elucidates the comparable clinical outcomes observed between the TBHC and EBHC cohorts in our study. Another study also demonstrated that catheter tips positioned in the frontal region yielded superior surgical outcomes in a single burr craniostomy with closed-system drainage and irrigation (25). The recurrence rates were 5% in the frontal region, 38% in the parietal, 36% in the occipital, and 33% at the temporal base. In our investigation, the majority of patients in the EBHC group had catheter tips situated in the occipital region, whereas patients in the TBHC group had tips located in both the occipital and frontal regions, which mitigated the incidence of recurrence. This disparity may contribute to the comparable outcomes observed between the TBHC and EBHC cohorts.

In our surgical practice, the endoscopic surgery allows surgeons to achieve more complete evacuation of hematomas, fibrous septa, and neovessels compared to TBHC. Consistent with clinical practice, our findings reveal that EBHC achieves a markedly higher hematoma clearance rate compared to TBHC, both at the first postoperative day and at discharge, underscoring the efficacy of endoscopic techniques in promoting hematoma evacuation. The superior efficacy of endoscopic visualization stems from its capacity to map fibrous septations’ 3D architecture, enabling targeted septotomy; whereas TBHC’s blind irrigation often leaves residual hematoma in obscured areas lacking real-time visual guidance. Due to the higher clearance rate associated with EBHC, patients often have an earlier drain removal time postoperatively. Our study revealed that the duration of head drainage was markedly reduced in the EBHC group compared to the TBHC group, averaging 18.66 ± 5.89 h for EBHC vs. 55.87 ± 23.03 h for TBHC. At 30 days postoperatively, the mortality rates exhibited no significant difference between the EBHC group and the TBHC group (4.2% vs. 2.6%; *p* = 0.680). The complication rates were recorded at 22.9% for the EBHC group and 15.4% for the TBHC group, respectively. Although the absolute number of patients experiencing complications was greater in the EBHC cohort, the difference was statistically insignificant (*p* > 0.05).

We also found that medical consumption among patients in the EBHC group was significantly higher compared to that in the TBHC group. TBHC is generally conducted under local anesthesia, whereas EBHC typically necessitates general anesthesia, potentially resulting in shorter operative durations and reduced healthcare expenditures in the TBHC group. In addition to the mode of anesthesia, multiple factors may contribute to the cost disparity between the EBHC and TBHC groups. The elevated costs associated with the EBHC group are likely driven by the utilization of specialized endoscopic equipment, prolonged operative times, heightened postoperative care requirements, increased risk of complications, and hospital stay duration. We also acknowledge that EBHC offers significant clinical advantages, including: (a) complete evacuation of the hematoma and excision of the fibrous membrane under direct visualization, (b) precise catheter insertion under direct visual guidance, minimizing the risk of brain or vascular injury, (c) real-time detection of acute bleeding events, identification of the bleeding source, and effective hemostasis, and (d) enhanced surgical depth and an expanded visual field (3). Therefore, EBHC may be more suitable for patients presenting with thick, organized septations that require direct visualization for effective septal lysis, or active neovessels detected on MRI, which demand precise endoscopic coagulation. Future investigations should incorporate these variables to comprehensively assess the cost-effectiveness of EBHC in comparison to TBHC for managing sCSDH. Additionally, the average duration of hospitalization was significantly extended in the EBHC group relative to the TBHC group. This disparity may be attributed to the fact that endoscopic procedures are generally more invasive than burr hole surgeries, which often require extended hospitalization and more comprehensive rehabilitation.

In our study, we found that in the EBHC group, 7 patients (14.6%) received antiplatelet therapy, and 5 patients (10.4%) were on anticoagulant therapy. In contrast, the TBHC group had 16 patients (41.0%) receiving antiplatelet therapy and 14 patients (35.9%) on anticoagulants. Furthermore, among the 48 patients in the EBHC group, 5 (10.4%) had a history of ischemic heart disease, compared to 11 of the 39 patients (28.2%) in the TBHC group (*p* = 0.033). These results suggest that our physicians are more likely to opt for the TBHC procedure for sCSDH, which typically involves a higher incidence of local anesthesia, and less surgical trauma, when treating patients who are long-term anticoagulant or antiplatelet users and those with heart disease. Additionally, we noted a significant difference in operative time. The mean operative time for the EBHC group was 103.56 ± 20.93 min, compared to 50.77 ± 12.40 min for the TBHC group (*p* < 0.001), underscoring the increased complexity and technical demands of endoscopic procedures. Endoscopic surgery necessitates specialized training and experience, and surgeons still on the learning curve for this approach may require extended operative times. Furthermore, the use of general anesthesia in the EBHC group may contribute to the extended operative duration.

Several limitations should be acknowledged in this study, including its single-center, retrospective design and the relatively modest sample size. These factors may constrain the generalizability of the findings. Future studies should prioritize multi-center, large-sample, prospective randomized controlled trials to provide more robust validation. Furthermore, surgeon expertise and institutional resources (e.g., endoscopy availability) heavily influence the choice of surgical techniques and outcomes for sCSDH. Centers with endoscopic proficiency may prioritize EBHC for complex cases, whereas TBHC remains a pragmatic choice in resource-limited settings. In future research, we aim to establish standardized criteria for selecting between different surgical approaches. To minimize bias, we will also implement standardized operative steps for each surgical approach.

Despite comparable recurrence rates, morbidity rates, and complication incidences observed between the TBHC and EBHC cohorts, it is noteworthy that TBHC demonstrated superior performance over EBHC in terms of mean operative time, medical resource consumption, and length of hospital stay in the treatment of sCSDH.

Therefore, we recommend TBHC technique to be widely used for sCSDH to avoid large craniotomies, particularly in elderly patients.

## Data Availability

The original contributions presented in the study are included in the article/supplementary material, further inquiries can be directed to the corresponding authors.
